# Exome-Wide Association Analysis of Coronary Artery Disease in the Kingdom of Saudi Arabia Population

**DOI:** 10.1371/journal.pone.0146502

**Published:** 2016-02-05

**Authors:** Carolien G. de Kovel, Flip Mulder, Jessica van Setten, Ruben van ‘t Slot, Abdullah Al-Rubaish, Abdullah M. Alshehri, Khalid Al Faraidy, Abdullah Al-Ali, Mohammed Al-Madan, Issa Al Aqaili, Emmanuel Larbi, Rudaynah Al-Ali, Alhusain Alzahrani, Folkert W. Asselbergs, Bobby P. C. Koeleman, Amein Al-Ali

**Affiliations:** 1 Department of Genetics, University Medical Center Utrecht, Utrecht, The Netherlands; 2 Department of Medicine, King Fahd Hospital of the University, University of Dammam, Dammam, Kingdom of Saudi Arabia; 3 Department of Cardiology, King Fahd Military Medical Complex, Al-Khobar, Kingdom of Saudi Arabia; 4 Prince Sultan Cardiac Center, Al-Ahssa, Kingdom of Saudi Arabia; 5 King Fahd Hospital of the University, University of Dammam, Dammam, Kingdom of Saudi Arabia; 6 Department of Medicine, Qatif Central Hospital, Qatif, Kingdom of Saudi Arabia; 7 College of Applied Medical Sciences, King Saud University, Riyadh, Kingdom of Saudi Arabia; 8 Department of Cardiology, Division Heart & Lungs, University Medical Center Utrecht, Utrecht, The Netherlands; 9 Prince Mohammed Center for Research & Consultation Studies, College of Medicine, University of Dammam, Dammam, Kingdom of Saudi Arabia; University of Texas, UNITED STATES

## Abstract

Coronary Artery Disease (CAD) remains the leading cause of mortality worldwide. Mortality rates associated with CAD have shown an exceptional increase particularly in fast developing economies like the Kingdom of Saudi Arabia (KSA). Over the past twenty years, CAD has become the leading cause of death in KSA and has reached epidemic proportions. This rise is undoubtedly caused by fast urbanization that is associated with a life-style that promotes CAD. However, the question remains whether genetics play a significant role and whether genetic susceptibility is increased in KSA compared to the well-studied Western European populations. Therefore, we performed an Exome-wide association study (EWAS) in 832 patients and 1,076 controls of Saudi Arabian origin to test whether population specific, strong genetic risk factors for CAD exist, or whether the polygenic risk score for known genetic risk factors for CAD, lipids, and Type 2 Diabetes show evidence for an enriched genetic burden. Our results do not show significant associations for a single genetic locus. However, the heritability estimate for CAD for this population was high (*h*^*2*^ = 0.53, S.E. = 0.1, p = 4e^-12^) and we observed a significant association of the polygenic risk score for CAD that demonstrates that the population of KSA, at least in part, shares the genetic risk associated to CAD in Western populations.

## Introduction

Coronary Artery Disease (CAD) is the major cause of death in most countries and has been reported to be responsible for 32% of deaths in Western populations [1]. Life style has been recognized as a major factor for the susceptibility to CAD and lack of exercise, smoking, and a diet of energy-dense fast food are the most important predisposing factors [2–4].

In addition, a large number of studies have shown that genetic risk factors play an important role in CAD [5–7]. These genetic risk factors can be divided in rare variants in genes with a strong effect, and common variants conferring low risk for disease. As expected, the rare mutations have been detected in several patients affected with a rare (<0.1% population frequency) single gene disorder [8,9]. These mutations usually confer high risk for a distinct and easily recognizable cardiac phenotype. At the other end of the spectrum, very common (10–50% population frequency) low risk variation has been found that associates to susceptibility for CAD. These common risk factors have been detected through genome-wide association studies (GWAS) of CAD and related phenotypes, such as blood lipids, obesity, and Type 2 Diabetes (T2D) [6, 10–13]. One of the first GWAS of CAD detected the 9p21 risk locus, centered on the single nucleotide polymorphism rs4977574. The risk allele has a population frequency in Caucasian populations of 49% and estimates of its risks vary between a relative risk (RR) of 1.29 and 1.39. Despite extensive fine mapping and functional studies the precise mechanism through which this locus predisposes to CAD remains unknown. Nevertheless, three genes in this region remain the most likely and interesting candidate genes, *CDKN2A CDKN2B*, and *ANRIL*. Furthermore, further association studies suggested that the 9p21 locus is associated to atherosclerosis underlying CAD, rather than acute Myocardial Infarction (MI). In contrast, a long suspected association between CAD and ABO blood group was confirmed to be linked to increased von Willebrand factor halflife, which in turn leads to increased risk for coronary thrombosis and MI. These early successes of GWAS have led to the formation of very large consortia that combine different GWAS data sets for mega- or meta-analysis, such as CARDIoGRAMplusC4D, which is composed of a case-control sample size of more than 240,000 subjects. These large studies have together reliably identified 52 risk loci that predispose to CAD and its different subphenotypes, all with convincing genome-wide significance and replication in independent datasets and populations [6]. However, these studies have largely been performed on Caucasian populations of European origin and no large association studies have been performed in Kingdom of Saudi Arabia (KSA) to assess the genetic risk specific for this population.

The rate of CAD and associated mortality has increased exceptionally over the past decades in the KSA [14]. This increase coincided with fast economic growth and urbanization that promotes sedentary life style, smoking, and a diet high in energy-dense fast food and low in fruits and vegetables [15]. These factors have undoubtedly contributed to the epidemic of CAD in KSA. Prior to its economic growth, KSA was a country that experienced scarcity. It may therefore be that the KSA population has genetic adaptations to a frugal lifestyle. Such adaptations may lead to increased susceptibility to CAD when exposed to a Western diet and lifestyle. It has been shown that natural selection can explain different population histories and lead to genetic heterogeneity of susceptibility to complex diseases [16]. One of the clearest examples is that different population frequency of HLA-alleles predisposing to Type 1 Diabetes coincides with population prevalence of disease [17–19]. Therefore, the question remains whether adaptation of the KSA population has led to an enrichment of specific genetic variation that promotes survival in frugal conditions, but that predispose to CAD in current KSA society.

To address this question we performed an EWAS of CAD in the KSA population to test the hypothesis that KSA population is enriched for genetic risk factors for CAD. For this purpose we used the Illumina Exome beadchip array and imputated missing genotypes to cover all known CAD associated genes, and include all rare and nonsynonymous variants. We tested whether specific strong genetic risk factors for CAD exist, or whether the polygenic risk score for known genetic risk factors for CAD, lipids, and T2D show evidence for an enriched genetic burden.

## Results

### Quality control and imputation results

We sampled 866 patients suffering from CAD of whom related phenotypes were available, including T2D and lipid profiles. Furthermore, we sampled 1,150 controls that were randomly selected from five major hospitals in the Eastern Province of KSA. Patient and control subjects were originating from the 13 provinces of the Kingdom, which was recorded and included in the QC analysis.

The samples were genotyped using the Illumina Infinium HumanExome BeadChip v1.1. This chip provides focused coverage for putative functional exonic variants and common Single Nucleotide Polymorphisms (SNPs) that are previously associated to CAD, metabolic syndrome and T2D. Rigorous quality control (QC) was performed after genotyping to remove genotyping errors and other sources of possible confounding. The majority of SNPs (53%) had very low minor allele frequency (MAF; less than 1%) or were monomorphic. Of the remaining SNPs, 74% had a MAF of more than 5% that is suitable for association mapping. To increase genomic coverage, we performed imputation resulting in more than 5 and 1.5 million SNPs with info-scores above 0.5 and 0.8, respectively. We performed association testing with genotyped SNPs only and with imputed SNPs included the two for info-score criteria to control spurious association. Further QC on sample identity and quality excluded another 108 subjects resulting in 832 cases and 1,076 controls for analysis (details of the QC procedure are provided in method section).

**Fig 1:**
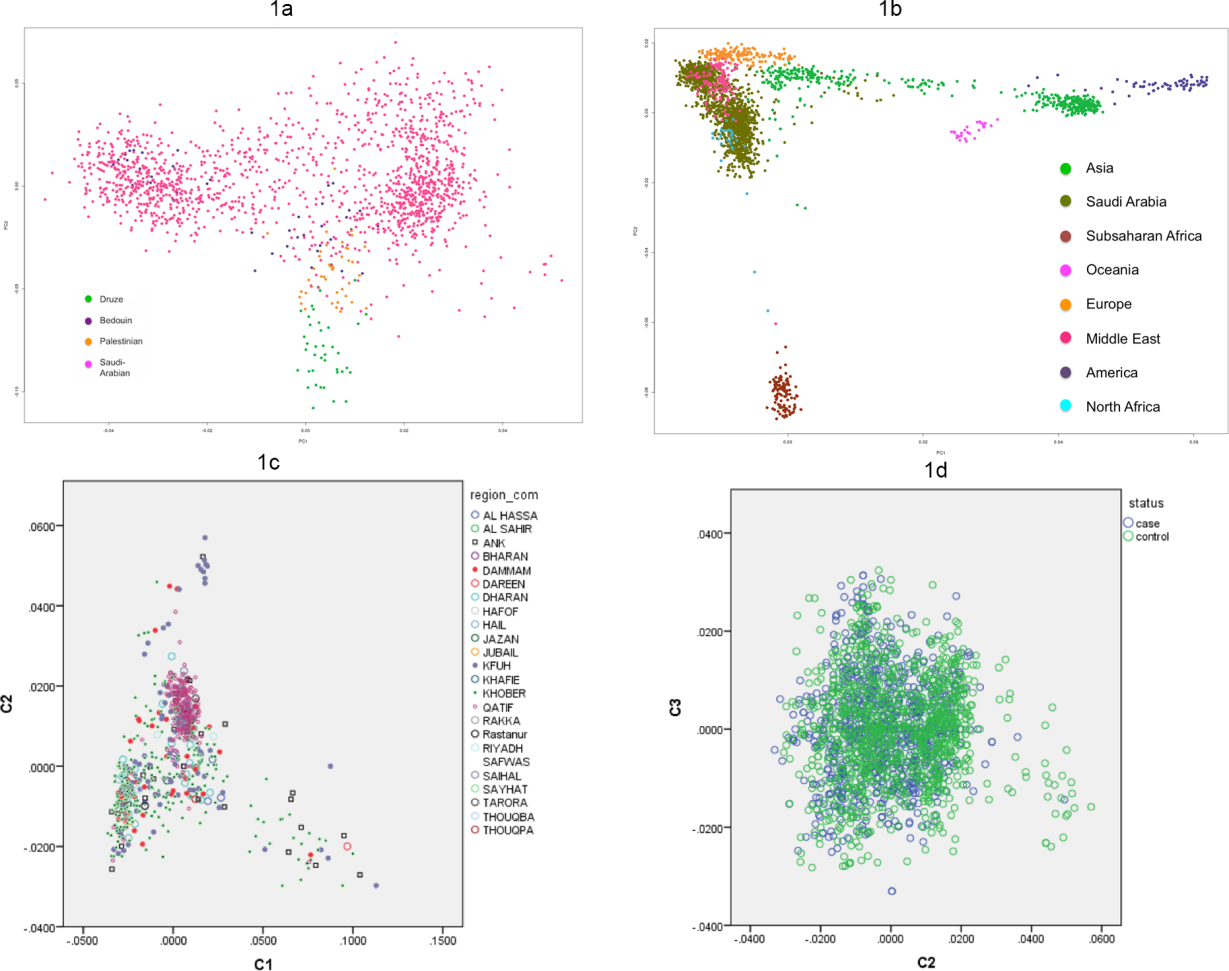
Study subjects (white diamonds) plotted with population reference samples for first (x-axis, PC1) and second (y-axis, PC2) principle components. (A)The clustering with Middle East populations, and (B) with main global populations as sampled in HGDP. (C) Study control subjects plotted according to the KSA province of origin, and (D) all KSA samples according to affection status for the most discriminating principle components.

Principle component analysis (PCA) was performed on non-imputed SNPs and the Human Genome Diversity Project (HGDP) dataset as a reference to assess population stratification between cases and controls, and to show genetic overlap between KSA and other populations (Fig 1). The result shows that KSA population overlaps partly with other Middle East populations (Fig 1A), but clusters separately from non-Middle East populations (Fig 1B).

PCA in KSA subjects alone confirmed sufficient overlap between cases and controls. However, it also revealed significant population structure that correlated with the KSA provinces from where the subjects originated (Fig 1C). To focus on a possible bias due to population stratification between cases and controls, we also plotted the PC-component analysis for cases and controls (Fig 1D).

Cases showed sufficient overlap with controls, apart from 12 controls and one case that are outliers (more than 4 s.d. from the centroid of the main cluster). Anova analysis of C1-C5 showed that only PC2 significantly differs between groups (p = 3.0E-07). To assure complete control of structure, we calculated association with the first 10 PCs from the above analyses as covariates to account for the minor regional differences.

To increase genomic coverage, we performed imputation using IMPUTE2 on the 1000 Genomes Phase I b37 June 2014 reference set. We selected SNPs that have info-scores of at least 0.5 or 0.8 and reported MAF of 2% or higher. This resulted in 3,285,290 and 1,023,828 SNPs with info-score of 0.5 and 0.8, respectively that were available for association testing. These SNPs were distributed across chromosomes reflecting sufficient genome-wide coverage. Concordance of imputation with genotyped SNPs was high (>95%).

### Genetic association testing for CAD in KSA: testing for novel genetic risk factors

**Fig 2:**
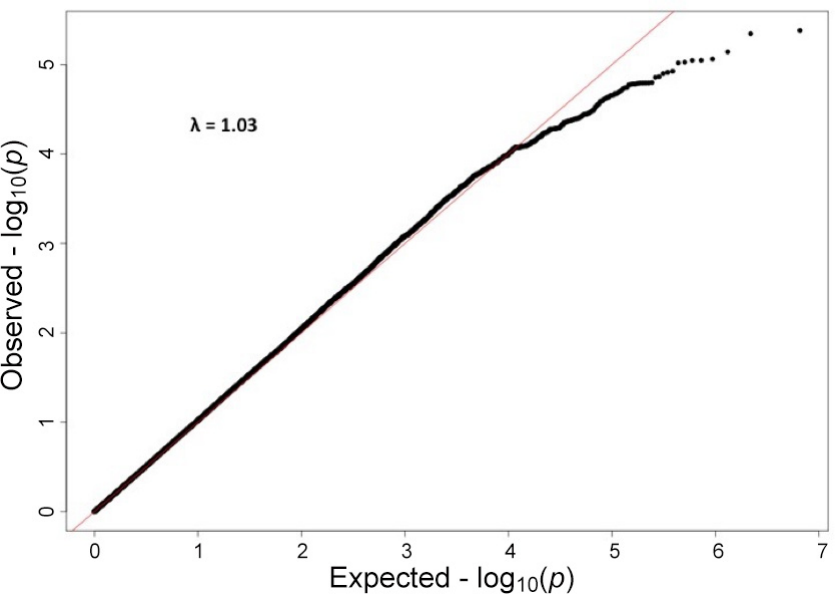
QQ-plots for association analysis. Figure shows the expected (x-axis) and observed (y-axis) log(p-values) for all SNPs with info-score > 0.5.

After QC, all remaining SNPs were tested for association with CAD. We have plotted the Quantile-Quantile plot, which showed effective control of population structure (λ **=** 1.03, QQ plot Fig 2). In these analyses, the tail of the QQ-curve representing more significant p-values seems to drop below the expected distribution, which may indicate overcorrection or the relative small sample size of our cohort for Exome-wide association.

**Fig 3:**
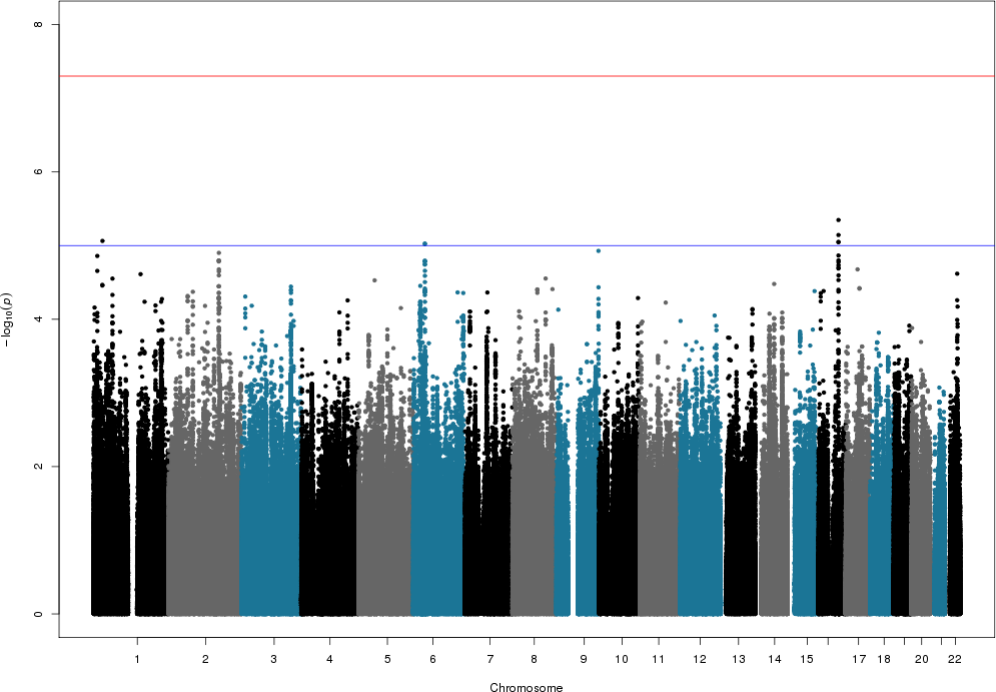
Manhattan plot for association testing of CAD in KSA population. The figure shows the p-value for association with disease (expressed as negative logarithm of p-value, y-axis) for each tested SNP, plotted against the chromosomal position of the SNP (x-axis). Figure shows the results for SNPs with info-scores > 0.5. Blue line indicates threshold for suggestive association, red line shows threshold for genome-wide significance.

Fig 3 shows the Manhattan plot for SNPs with info-scores >0.5. No SNP reached genome-wide significance indicating that no high-risk loci could be detected in this cohort. Three regions show suggestive evidence for association (p<1x10^-05^) for info-score > 0.5. These regions are on chromosomes 1 (in the *PTPRU* gene), 6 (*DNAH8* gene), 7 (*LHFPL3* gene), and 16 (upstream of *CAD5*). None of these regions are overlapping with the known CAD risk loci. Overall, the lack of genome-wide association suggests that the population of KSA does not carry an unknown population specific genetic factor conferring strong risk for CAD such that it can be detected in this cohort.

Finally, we calculated the association for genotyped SNPs using the FastLMM algorithm that is efficient in removing population structure, as well as cryptic relatedness. This association analysis, as expected, also did not result in any SNP exceeding the threshold of 5E-08. More importantly, the top SNPs did not differ significantly with those from the previous analysis suggesting that the logistic regression analyses are valid.

### Polygenic risk score: testing for an increased burden of known genetic risk factors for CAD

We collated a list of all risk SNPs of different CAD traits, including CAD, diabetes, and various serum lipid levels (LDL, HDL, TC and TG). Allele frequencies, identity, and odds ratios were taken from the GWAS database. This resulted in a list of 304 risk SNPs that were present in our data set. Table 1 below shows the resulting p-values. The score for CAD risk loci showed a significant p-value after correction for six tests (p = 0.0008; corrected-p = 0.005), suggesting that indeed the known CAD genetic risk factors contribute to CAD susceptibility in the KSA population. This observation suggests that the known risk loci have similar effect sizes in the KSA population and larger more powerful cohorts may result in confirmation of the individual known common small effect risk factors, and may allow identification of population specific factors with similar effect sizes.

**Table 1:** Polygenic risk score for CAD related traits. The table shows the number of known SNPs, number of SNPs present in the current data set (#SNPs included), and resulting p-value for the polygenic score.

Trait	# known SNPs	# SNPs included	p-value
T2D	37	29	0.32
HDL	72	50	0.94
LDL	58	42	0.06
TG	32	25	0.26
TC	53	36	0.74
CAD	52	31	0.0008

### Heritability analysis

We estimated the SNP-heritability for CAD phenotype using the GCTA tool and the genome-wide genotyped SNP data, including the 31 genotyped SNPs that are known to be associated with CAD. Using the genotyped SNPs, we estimated that 0.13 (SE 0.03) of the phenotypic variance could be explained by genetic factors out of a total of 0.24 (SE 0.009) phenotypic variance observed. These calculations provide a narrow sense heritability estimate of *h*^*2*^ = 0.53 (S.E. = 0.1) (p = 4e^-12^) for this population. This estimate is at the higher bound of what has been estimated for Western European Caucasian populations (*h*^*2*^ = 0.39 (S.E = 0.06))(6).

## Discussion

We present here the first Exome-wide association study of CAD in the population of KSA. Several studies of various CAD traits in populations of mainly Western European ethnicity have demonstrated that a large number of common low risk loci exist. The most remarkable locus is at chromosome 9p21, which consist of a number of associated SNPs in high linkage disequilibrium with each other and spans the *CDK2BAS* or *ANRIL* gene, which is a non-protein coding RNA. Even though the chromosome 9p21 association is the most pronounced CAD association, its effect size is still relatively modest, yet falls within the range of detectable loci of our current study (Odds Ratio of ~1.45 for heterozygotes, 1.9 for homozygotes). However, we were unable to detect even suggestive association with the 9p21 region, which may indicate that the risk conferred by this locus is less strong than anticipated and that other unknown disease mechanisms may play a role in CAD in KSA.

Apart from this locus, 52 other risk loci for CAD traits with more modest effect sizes have been detected previously. The magnitude of the risk conferred by these loci individually fluctuate between OR of 0.8 to 1.2 [6, 20, 21]. Such low risk is not expected to be detected in our limited sample size, and indeed no suggestive association for any of the known risk loci was observed. Apart from the magnitude of effect size, our study focused on variations in, or near previous associated genes. Therefore, we may have not reached optimal coverage for risk loci mapping in intergenic regions. However, the majority of associated SNPs were directly genotyped in our study and did not show genome-wide significant evidence for association. Given our limited sample size, this result suggests the known risk loci detected in Western Caucasian populations also confer similar low risk for disease in KSA. We conclude that our study suggests that of the known CAD risk loci, none confer higher risk to the KSA population and therefore these can not explain the high prevalence of CAD in the KSA population.

We proceeded to calculate polygenic risk scores for CAD and CAD risk factors, to test for an excess or accumulation of the multiple known genetic risk loci. We detected significant association for the CAD risk score. This result suggests that the known genetic risk loci for CAD also contribute to the risk of CAD in the KSA population. As our study is modest in size compared to other international cohorts, we expect that an increase of our sample size will enable us to detect significance for known and novel ‘KSA’ risk factors.

We have performed careful quality control procedures to prevent spurious associations. Apart from the standard procedures that filter parameters that reflect genotyping accuracy and population stratification, we performed step-wise analyses including a ‘true’ genotyped SNP analysis only, and two analyses for imputed SNP with a different threshold for imputation quality (info-scores). Finally, we verified the effect of including correction for population stratification using principle components. We observed that correction for population stratification was indeed needed as it removed apparent inflation of our test statistic represented by ‘lambda’ scores above 1.1 calculated from the QQ-plots. The correct analysis including principle components showed indeed no evidence for inflation of the test statistics. Therefore, association with imputed SNPs showed validity and significantly increased the number of analyzed SNPs to more than 3 million SNPs, providing good coverage of all genes in the human genome. Nevertheless, we consent that our gene-centered study represents a ‘first look’ that has some blind spots. Therefore, increasing both the number of subjects and including additional genome-wide genotyping may uncover genetic associations that were unable to be detected in our current analysis.

In addition, the SNP content of Illumina genotyping platforms has been designed with sequencing data of mainly Western populations. The KSA population may differ significantly in type and frequency of exonic SNPs compared to Caucasian populations of European origin. Large scale sequencing studies of the KSA (diseased) population may therefore uncover population specific variations that could be missed in this study. Nevertheless, it is expected that efficacy of SNPs selected for indirect association mapping in Caucasian populations is transferable to Middle Eastern populations [22].

Finally, our study provides a population genetics view of the KSA population. The principle component analysis using other Middle Eastern and worldwide populations showed that the KSA subjects cluster separately as a genetically distinct Middle Eastern population. Furthermore, we observed distinct clustering of subjects who were randomly selected from five major hospitals in the Eastern Province of KSA, where the patient groups are derived from the 13 provinces of the Kingdom. This undoubtedly reflects the demographic history of the KSA population, yet is not unexpected, as similar differences have been observed in the British population (WTCCC-study), and even in a country as small as The Netherlands (GoNL-study) [23,24].

## Conclusion

Our study demonstrates that the known genetic risk for CAD is similar in magnitude in KSA compared to previously studied populations. Furthermore, it is unlikely that population specific common genetic risk factors with large effects exist in the KSA population. Our study is, however, relatively small in size as compared to contemporary GWAS studies and power to detect low risk SNPs is therefore limited. Controls were well matched for regional differences, as is evidenced by the low genomic control inflation (lambda) scores that reflect the degree of population stratification. Controls were however much younger in age than cases. Consequently, it is expected that a significant proportion our controls will become affected over time, further reducing power. Therefore, to successfully map the genetic variants predisposing to CAD in KSA, second phase studies equal in size to contemporary national CAD studies are needed. Finally, it is likely that the rare exonic variants in the KSA population are different in type and frequency than those present in Western populations. Therefore, large scale sequencing studies of the KSA (diseased) population are needed to uncover population specific variations that may be of great relevance for disease.

## Methods

### Patients

**Fig 4:**
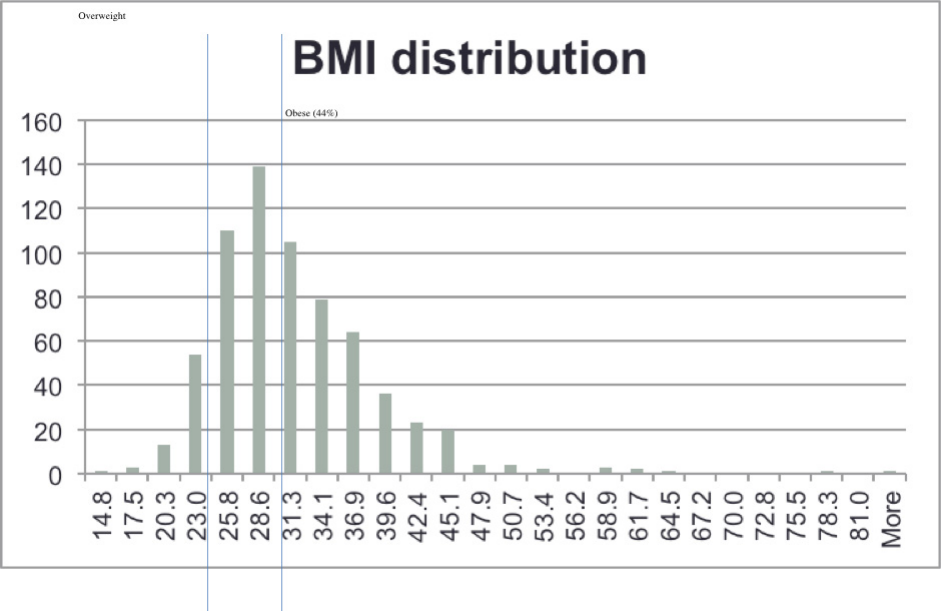
Distribution of BMI. X-axis gives BMI, y-axis shows number of subjects.

The participants provided written informed consent. The written informed consent forms have all been documented electronically against each participants name and data. The ethics committee approved the project and the consent procedure. This study was approved by the ethical board of the University of Damman. All patients and controls were randomly selected from five major hospitals in the Eastern Province of KSA, where the patient groups are derived from the 13 provinces of the Kingdom. DNA was extracted from peripheral blood using standard methods. In total 866 CAD patients (321 female, 531 male, 14 gender unknown) and 1,150 control individuals (235 female, 847 male, 68 gender unknown) were collected for genotyping. The majority of patient cases had experienced a myocardial event (98%) and 672 (78%) had diabetes. In addition, the majority of patient cases were overweight as defined by a BMI above 25 (520, 77%), and 292 (43%) were obese (BMI greater than 30) (Fig 4).

**Fig 5:**
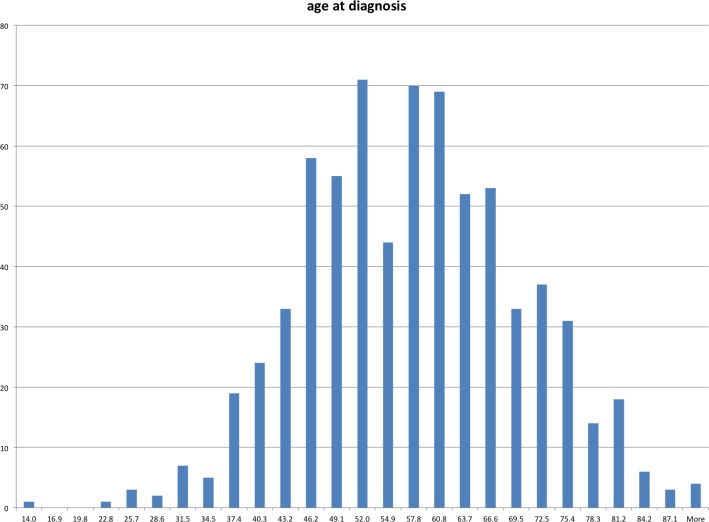
Distribution of age at diagnosis. X-axis shows age, y-axis number of subjects.

For 675 patients the number of myocardial events was recorded, of which 213 (32%) had experienced two or more events. For 713 patients the age at diagnosis (AAD) was determined and followed a normal distribution (Fig 5). Median AAD was 56 years of age, (lowest was 24 and highest was 90 years).

### Genotyping

The Illumina Infinium HumanExome Beadchip v1.1 was used on the Illumina iScan according to manufacturer’s specifications. The HumanExome Beadchip provides excellent coverage of putative functional variants in or near the coding region of the human genome. The exonic content provides variants representing a wide diversity of populations and is not focused on Caucasian samples. The chip contains 260,575 SNPs located within 10 kb of RefSeq genes, of which the majority (258,304) are located within the coding sequences. Finally, 232,125 SNPs are nonsynonymous SNPs that represent likely functional variants.

Many SNPs have low minor allele frequency (MAF) and 127,137 genotyped SNPs were monomorphic in our cohort. We genotyped the 2,016 samples on 168 Human Exome Beadchips (12 samples per Beadchip, seven kits of 288 samples each (kit number: WG-353-1105).

### Quality Control and imputation of genotypes

Rigorous quality control was applied to remove spurious signal due to confounding. We used the following parameters for exclusion of subjects: i) individuals with less than 95% of SNPs successfully genotyped; ii) samples with IBD values indicating relatedness (IBD > 1.5 were considered duplicates (88 pairs), IBD > 0.9 as being first degree relatives (only one individual of each family was included in the analysis, 29 pairs); iii) individuals showing heterozygosity scores deviating more than six standard deviations from the mean were excluded; iv) markers with call rates of 95% or less were excluded; v) markers with allele distributions strongly deviating from Hardy-Weinberg (HW) equilibrium in controls (p-value < 1x10E-05) were discarded (step iii-v resulted in 75 subjects excluded). This resulted in 832 cases and 1,076 controls for analysis.

This sample was used for imputation of untyped SNPs with IMPUTE2 and the 1000 Genomes Phase I reference panel. Prephasing was performed on divided regions of less than 200 markers not spanning the centromere or ends of chromosome. IMPUTE was run using best-guess haplotypes. Imputed SNPs were filtered for SNPs not passing our QC criteria as outlined above. SNP imputation showed an accuracy of 97.89%. Principle component analysis was performed using Plink and 4,000 selected SNPs that are present in Hapmap, which were pruned from the total set using r^2^<0.001. Imputation was automated using an in-house pipeline that was written as a combination of bash and R scripts that call the following tools: Plink, SNPTest, QCTool, SHAPEIT, and IMPUTE2 [25–28]. First, the raw genotypes per chromosome are run. The 'liftover' tool removes unmapped markers, and QC as described above was conducted. All AT/CG markers are removed, as they are rarely included in Illumina genotyping chips and are prone to bias due to allele misidentification. Flip strand errors were checked, duplicate markers removed and phasing was done using SHAPEIT on the 1000 Genomes Phase I b37 June 2014 reference.

Chromosomes were divided in chunks of minimal 5 MB in size containing at least 200 markers and were imputed using IMPUTE2 (1000 Genomes Phase I b37 June 2014 reference) in a pipeline validating correct processing.

After imputation, each chunk was processed through QCTool to filter out all markers not passing our criteria (info-score < 0.5 or 0.8, MAF < 0.05, HWE p-value < 1x10E-06, and SNP missing < 0.05).

### Statistical analysis

We performed logistic regression association testing using SNPtest software, using frequentist method expected with and without the first ten covariates from the PC-analysis. Manhattan and QQ-plots were generated by in-house R scripts. Genome-wide distribution of the test statistic indicated no evidence for population stratification (QQ plots). We used the benchmark levels of significance for declaring significant association (p<5x10^-08^) and suggestive association (p<1x10^-05^).

Analysis of Variance (ANOVA) of principle components (PCs) was used to test for significant difference of these PCs between cases and controls, using R scripting.

Risk profiles were computed by PLINK using the GRS option with the risk alleles and beta-values (log(OR)) provided. Differences in risk scores between cases and controls were tested with two-sided t-test with assumed equal variance.

SNP- heritability was estimated using the GCTA tool implemented in the Plink software. The analysis model included affection status and non-imputed genotypes as covariates. The genetic similarity threshold was set to 0.05.

Summary data of the results is available at: http://www.gwascentral.org/study/HGVST1833, and genotype data at The European Genome-phenome Archive (EGA) as study "CVD-KSA".

### Power

**Fig 6:**
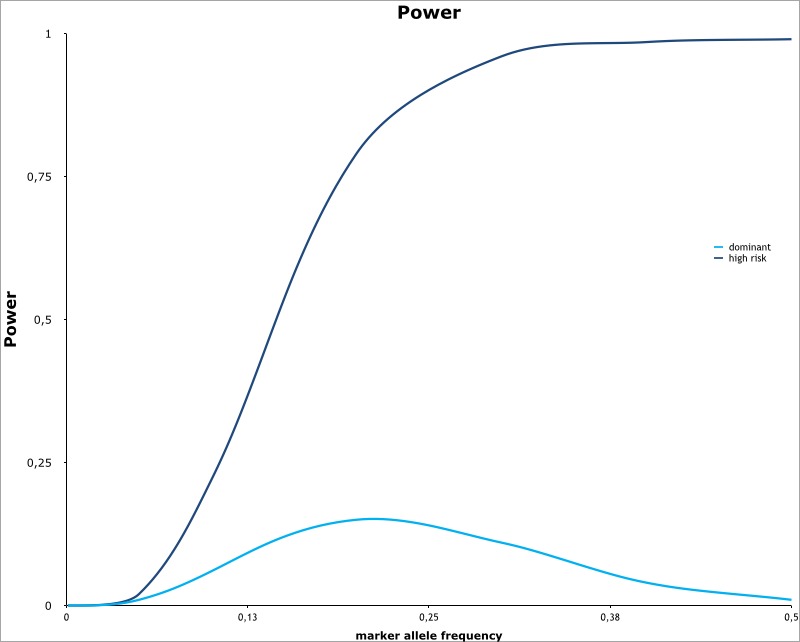
Power of the study.

Power was calculated as the probability to detect association in the use cohort with threshold of significance p = 10^−7^ (Fig 6). We calculated power for the 'high risk' hypothesis that strong risk (genotype relative risk = 1.5 for heterozygotes and 2.5 for homozygotes) may underlie CAD risk in KSA. As a comparison, we also calculated power for the standard dominant model using relative risk of 1.5 for both heterozygous and homozygous genotypes. The results show that our study has only sufficient power (>80% when MAF > 20%) to detect such a high risk factor if it follows an additive inheritance model. So far, known risk factors for CAD have lower risks and follow a dominant or non-additive model of inheritance. Our power calculation shows that our study has less than 20% power to detect such loci.

## URL

PLINK software:

http://pngu.mgh.harvard.edu/purcell/plink/

SNPTest, ShapeIt, QCTools, and Impute2:

http://www.stats.ox.ac.uk/~marchini/software/gwas/gwas.html

GWAS database:

http://www.genome.gov/gwastudies/index.cfm?#searchForm

Link to summary data of this study:

http://www.gwascentral.org/study/HGVST1833

## References

[1] van der Bom T, Zomer AC, Zwinderman AH, Meijboom FJ, Bouma BJ, Mulder BJ. The changing epidemiology of congenital heart disease. Nat Rev Cardiol. 2011;8(1):50–60. 10.1038/nrcardio.2010.166 21045784

[2] Chow CK, Lock K, Teo K, Subramanian SV, McKee M, Yusuf S. Environmental and societal influences acting on cardiovascular risk factors and disease at a population level: a review. Int J Epidemiol. 2009;38(6):1580–94. 10.1093/ije/dyn258 19261658 PMC2786248

[3] O'Toole TE, Conklin DJ, Bhatnagar A. Environmental risk factors for heart disease. Rev Environ Health. 2008;23(3):167–202. 19119685 10.1515/reveh.2008.23.3.167

[4] Levenson JW, Skerrett PJ, Gaziano JM. Reducing the global burden of cardiovascular disease: the role of risk factors. Prev Cardiol. 2002;5(4):188–99. 12417828 10.1111/j.1520-037x.2002.00564.x

[5] Miller CL, Assimes TL, Montgomery SB, Quertermous T. Dissecting the causal genetic mechanisms of coronary heart disease. Curr Atheroscler Rep. 2014;16(5):406 10.1007/s11883-014-0406-4 24623178 PMC4015632

[6] CARDIoGRAMplusC4D Consortium, Deloukas P, Kanoni S, Willenborg C, Farrall M, Assimes TL, et al Large-scale association analysis identifies new risk loci for coronary artery disease. Nat Genet. 2013;45(1):25–33. 10.1038/ng.2480 23202125 PMC3679547

[7] Gelb BD, Chung WK. Complex Genetics and the Etiology of Human Congenital Heart Disease. Cold Spring Harb Perspect Med. 2014;4(7). 10.1101/cshperspect.a013953 PMC4066638 24985128

[8] Yuan S1, Zaidi S, Brueckner M. Congenital heart disease: emerging themes linking genetics and development. Curr Opin Genet Dev. 2013;23(3):352–9 10.1016/j.gde.2013.05.004 23790954 PMC4154700

[9] Fahed AC, Gelb BD, Seidman JG, Seidman CE. Genetics of congenital heart disease: the glass half empty. Circ Res. 2013;112(4):707–20. 10.1161/CIRCRESAHA.112.300853 23410880 PMC3827691

[10] International Consortium for Blood Pressure Genome-Wide Association Studies, Ehret GB, Munroe PB, Rice KM, Bochud M, Johnson AD, et al Genetic variants in novel pathways influence blood pressure and cardiovascular disease risk. Nature. 2011;478(7367):103–9. 10.1038/nature10405 21909115 PMC3340926

[11] Teslovich TM, Musunuru K, Smith AV, Edmondson AC, Stylianou IM, Koseki M, et al Biological, clinical and population relevance of 95 loci for blood lipids. Nature. 2010 8 5;466(7307):707–13. 10.1038/nature09270 20686565 PMC3039276

[12] Speliotes EK, Willer CJ, Berndt SI, Monda KL, Thorleifsson G, Jackson AU, et al Association analyses of 249,796 individuals reveal 18 new loci associated with body mass index. Nat Genet. 2010;42(11):937–48. 10.1038/ng.686 20935630 PMC3014648

[13] DIAbetes Genetics Replication And Meta-analysis (DIAGRAM) Consortium; Asian Genetic Epidemiology Network Type 2 Diabetes (AGEN-T2D) Consortium; South Asian Type 2 Diabetes (SAT2D) Consortium; Mexican American Type 2 Diabetes (MAT2D) Consortium; Type 2 Diabetes Genetic Exploration by Nex-generation sequencing in muylti-Ethnic Samples (T2D-GENES) Consortium, Mahajan A, et al Genome-wide trans-ancestry meta-analysis provides insight into the genetic architecture of type 2 diabetes susceptibility. Nat Genet. 2014;46(3):234–44. 10.1038/ng.2897 24509480 PMC3969612

[14] Mokdad AH, Jaber S, Aziz MI, AlBuhairan F, AlGhaithi A5, AlHamad NM, et al The state of health in the Arab world, 1990–2010: an analysis of the burden of diseases, injuries, and risk factors. Lancet. 2014;383(9914):309–20. 10.1016/S0140-6736(13)62189-3 24452042

[15] Mehio Sibai A, Nasreddine L, Mokdad AH, Adra N, Tabet M, Hwalla N. Nutrition transition and cardiovascular disease risk factors in Middle East and North Africa countries: reviewing the evidence. Ann Nutr Metab. 2010;57(3–4):193–203. 10.1159/000321527 21088386

[16] Li M, Luo XJ, Rietschel M, Lewis CM, Mattheisen M, Müller-Myhsok B, et al Allelic differences between Europeans and Chinese for CREB1 SNPs and their implications in gene expression regulation, hippocampal structure and function, and bipolar disorder susceptibility. Mol Psychiatry. 2014;19(4):452–61. 10.1038/mp.2013.37 23568192 PMC3937299

[17] Kukko M, Virtanen SM, Toivonen A, Simell S, Korhonen S, Ilonen J, et al Geographical variation in risk HLA-DQB1 genotypes for type 1 diabetes and signs of beta-cell autoimmunity in a high-incidence country. Diabetes Care. 2004;27(3):676–81. 14988284 10.2337/diacare.27.3.676

[18] Dorman JS, LaPorte RE, Stone RA, Trucco M. Worldwide differences in the incidence of type I diabetes are associated with amino acid variation at position 57 of the HLA-DQ beta chain. Proc Natl Acad Sci U S A. 1990;87(19):7370–4. 2217170 10.1073/pnas.87.19.7370 PMC54748

[19] Borchers AT, Uibo R, Gershwin ME. The geoepidemiology of type 1 diabetes. Autoimmun Rev. 2010;9(5):A355–65. 10.1016/j.autrev.2009.12.003 19969107

[20] Schunkert H, Konig IR, Kathiresan S, Reilly MP, Assimes TL, Holm H, et al Large-scale association analysis identifies 13 new susceptibility loci for coronary artery disease. Nat Genet 2011;43:333–338. 10.1038/ng.784 21378990 PMC3119261

[21] Ozaki K, Tanaka T. Molecular genetics of coronary artery disease. J Hum Genet. 2015 Epub. 10.1038/jhg.2015.70 26134515

[22] Gonzalez-Neira A, Ke X, Lao O, Calafell F, Navarro A, Comas D, et al The portability of tagSNPs across populations: A worldwide survey. Genome Res 2006;16:323–330.Purcell S, Neale B, Todd-Brown K, Thomas L, Ferreira MAR, Bender D, et al. PLINK: a toolset for whole-genome association and population-based linkage analysis. American Journal of Human Genetics 2007; 81. 16467560 10.1101/gr.4138406 PMC1415211

[23] Genome of the Netherlands Consortium. Whole-genome sequence variation, population structure and demographic history of the Dutch population. Nature Genetics 2014;46:818–25. 10.1038/ng.3021 24974849

[24] Wellcome Trust Case Control Consortium Genome-wide association study of 14,000 cases of seven common diseases and 3,000 shared controls. Nature 2007;447:661–678. 17554300 10.1038/nature05911 PMC2719288

[25] Purcell S, Neale B, Todd-Brown K, Thomas L, Ferreira MAR, Bender D, et al PLINK: a toolset for whole-genome association and population-based linkage analysis. American Journal of Human Genetics 2007; 81. 10.1086/519795 PMC1950838 17701901

[26] Marchini J, Howie B. Genotype imputation for genome-wide association studies. Nature Reviews Genetics 2010;11,499–511. 10.1038/nrg2796 20517342

[27] Delaneau O, Marchini J, Zagury J-F. A linear complexity phasing method for thousands of genomes. Nature Methods 2012;9,179–181. 10.1038/nmeth.1785 22138821

[28] Marchini J, Howie B, Myers S, McVean G, Donnelly P. A new multipoint method for genome-wide association studies by imputation of genotypes. Nature Genetics 2007;39:906–913. 17572673 10.1038/ng2088

